# Role of TFH Cells in Promoting T Helper 17-Induced Neuroinflammation

**DOI:** 10.3389/fimmu.2018.00382

**Published:** 2018-02-27

**Authors:** James L. Quinn, Gaurav Kumar, Agnieshka Agasing, Rose M. Ko, Robert C. Axtell

**Affiliations:** ^1^Department of Microbiology and Immunology, University of Oklahoma Health Sciences Center, Oklahoma City, OK, United States; ^2^Arthritis and Clinical Immunology Program, Oklahoma Medical Research Foundation, Oklahoma City, OK, United States

**Keywords:** experimental autoimmune encephalomyelitis, Th17, TFH, B cells, CXCL13, multiple sclerosis

## Abstract

Both T cells and B cells are implicated in the pathology of multiple sclerosis (MS), but how these cells cooperate to drive disease remains unclear. Recent studies using experimental autoimmune encephalomyelitis (EAE) demonstrated that the TH17 pathway is correlated with increased numbers of ectopic B-cell follicles in the central nervous system (CNS). As follicular T helper (TFH) cells are regulators of B cell responses, we sought to examine the role of TFH cells in EAE induced by the transfer of myelin-specific TH17 cells (TH17-EAE). In this study, we first confirmed previous reports that B-cells are a major cell type infiltrating the CNS during TH17-EAE. In addition, we found that B cells contribute to the severity of TH17-EAE. Class-switched B-cells in the CNS were positively correlated with disease and, strikingly, the severity TH17-EAE was diminished in B cell deficient mice. We next focused on the role TFH cells play in TH17-EAE. We found substantial numbers of CXCR5^+^PD1^+^CD4^+^ TFH cells in the CNS tissue of TH17-EAE mice and that at the peak of disease, the number of infiltrating TFHs was correlated with the number of infiltrating B-cells. Using congenic CD45.1^+^ donor mice and CD45.2^+^ recipient mice, we determined that the TFH cells were recipient-derived, whereas IL-17^+^ cells were donor-derived. We assessed whether myelin-specific TFH cells are capable of inducing EAE in recipient mice and found that transferring TFH cells failed to induce EAE. Finally, we tested the effects of blocking TFH trafficking in TH17-EAE using an antagonistic antibody against CXCL13, the chemokine ligand for CXCR5 on TFH cells. We found anti-CXCL13 treatment significantly reduced TH17-EAE disease. This treatment blocked CD4^+^ T cells from entering the CNS, but had no effect on infiltration of B cells. Strikingly, this antibody treatment had no measurable effect on TH17 disease in B cell-deficient mice. These data demonstrate that infiltrating TFH cells are a key cell type that contributes to an inflammatory B cell response in TH17-EAE and provide evidence for targeting TFH cells as a treatment for neuro-autoimmune diseases like MS.

## Introduction

Multiple Sclerosis (MS) is a neuro-inflammatory disorder, which results in the infiltration of immune cells into the central nervous system (CNS) and demyelination of neurons (1). CD4^+^ T helper (TH) cells play a critical role driving disease in both MS and the mouse model of the disease, experimental autoimmune encephalomyelitis (EAE) (2, 3). In fact, the transfer of myelin-specific T helper cells alone is capable of inducing EAE disease in healthy recipient mice. While both TH1 and TH17 cells are capable of inducing disease, each cell type results in a unique disease pathology. EAE induced by the transfer of myelin-specific TH17 cells (TH17-EAE) is characterized by elevated numbers of B cells and neutrophils in the CNS compared to TH1-induced EAE (3, 4). Additionally, TH17-EAE mice develop aggregates of B cells and T cells in the meninges of CNS tissue, also known as ectopic follicles (4).

The recent successes of several B cell-depleting therapies demonstrate B cells also play a critical role in MS and EAE disease progression (5–8). The mechanisms though which B cells lead to disease activity are threefold. First, plasma cells generate autoantibodies (9). Second, B cells produce inflammatory cytokines such as IL-6 (10). Finally, B cells can act as antigen presenting cells to T cells and help maintain an autoreactive T cell response (11). Although previous work has clearly demonstrated that both B cells and helper T cells contribute to disease progression, it is not currently clear if and how these two populations cooperate in MS.

CXCL13 is a chemokine that plays a critical role in the homing and co-localization of B cells and T cells to the follicles of lymphoid organs as well as the formation of ectopic follicles outside of lymphoid organs (12, 13). It has been shown in several disease models that a TH17 signature is associated with elevated levels of CXCL13 in the blood and tissue (14, 15). In EAE mice specifically, CXCL13 is elevated in TH17-EAE mice compared to actively immunized mice (4). Additionally, in MS patients, CXCL13 is significantly elevated in the peripheral blood and found in active lesions of the brain (16–18).

The primary receptor of CXCL13, CXCR5, is found on subsets of both B cells and T cells. The primary T cell subset that expresses CXCR5 is follicular T helper (TFH) cells (19). TFH cells have been well studied in the follicles of secondary lymphoid tissue in the context of infection, where they directly interact with proliferating B cells to aid in germinal center (GC) formation, affinity maturation, and maintenance of memory B cells (20). In MS patients, a recent study has shown a positive correlation between CXCR5^+^ TFH cell numbers in blood and increases in disability (21). Additionally, effective Laquinomod treatment of EAE mice was associated with decreased numbers of TFH cells and B cell responses (22). Though these reports show strong correlative data indicating that TFH cells may contribute to disease activity in MS and EAE, there is currently no study that definitively shows the causal effects of TFH cells in this disease.

The purpose of our study is to better understand the role of CXCR5^+^ TFH cells in autoimmune CNS inflammation. Our results demonstrate elevated levels of TFH cells in the CNS tissue of TH17-EAE mice. Additionally, we found that donor-derived TH17 cells first infiltrate the CNS and are followed by a second wave of infiltration, which include both TFH cells and B cells. These two populations are positively correlated and this relationship indicates that these TFH cells are promoting a pro-B cell environment within the CNS tissue. This role of TFH cells is further demonstrated by the reduction of TH17-EAE disease following anti-CXCL13 treatment. Additionally, we observed no significant effect on TH17-EAE in B cell-deficient mice treated with CXCL13 antibody. This shows an important role for CXCR5^+^ T cells in line with their known ability to support B cell function and begins to answer important questions regarding their contribution to disease progression.

## Materials and Methods

### Mice

C57BL/6 (WT, CD45.2^+^), B6.SJL-Ptprca Pepcb/BoyJ (CD45.1^+^), and B6.129S2-*Ighm^tm1Cgn^*/J (μMT) mice were purchased from Jackson Laboratory and bred in the Oklahoma Medical Research Foundation mouse facility. All animals were treated in compliance with the institutional guidelines. All experiments were performed using 8- to 10-week-old female mice.

### EAE Induction

For adoptive transfer EAE, age- and sex-matched donor mice were subcutaneously immunized with 150 µg MOG_35–55_ peptide (Genemed Synthesis, Inc.) emulsified in complete Freund’s adjuvant (5 mg/ml heat-killed *M. tuberculosis*), followed by an intraperitoneal (IP) injection of 250 ng of *Bordetella pertussis* toxin (List Biological Laboratories, Inc.) in 200 µl of PBS at 0 and 2 days postimmunization. Ten days postimmunization, spleens and lymph nodes were collected and mechanically disrupted to generate a single-cell suspension. For TH17-EAE, the cells were cultured at 2.5 × 10^6^ cells/ml for 72 h and stimulated with 10 µg/ml MOG_35–55_, 10 ng/ml IL-23, and 10 µg/ml IFN-γ antibody in complete RPMI media (23). For TFH-EAE, cells were cultured with 10 µg/ml MOG_35–55_, 20 ng/ml IL-6, 20 ng/ml IL-21, 10 µg/ml IFN-γ antibody, 10 µg/ml IL-4 antibody, and 20 µg/ml TGF-β antibody in complete RPMI media as previously described (24). On Day 3, cells were collected and 5 × 10^6^ cultured cells were transferred into healthy recipient mice by IP injection.

Mice were monitored daily for clinical signs. Paralysis was assessed using a standard clinical score ranging from 0 to 5 with scores corresponding to the following phenotypes: 0, no disease; 1, loss of tail tone; 2, partial hind-limb paralysis; 3, complete hind-limb paralysis; 4, forelimb paralysis; and 5, moribund/dead.

### Isolation of CNS-Infiltrating Cells

Cells were isolated from the brainstem, cerebellum, and spinal cords of PBS-perfused mice. CNS homogenates were incubated with 5 µl/mL DNAse (Sigma) and 4 mg/ml collagenase (Roche) at 37°C for 40 min. and purified using a Percoll (GE Healthcare) gradient.

### CXCL13 Antibody Treatment

Anti-mouse CXCL13 and isotype antibodies were provided by Dr. Maurice Zauderer (Vaccinex). Beginning on the day of transfer, mice were treated with 30 mg/kg of the antibodies in phosphate buffer saline, intraperitoneally, twice a week until sacrifice.

### Quantitative Real-time PCR

Following culture, CD4^+^ T cells were isolated using a magnetic CD4 negative enrichment kit (Miltenyi Biotec). Total RNA was extracted using the RNeasy Mini Kit (Qiagen) and reverse-transcribed into cDNA by iScript cDNA Synthesis Kit (Bio-Rad). Q-PCR was performed using iQ SYBR Green Supermix (Bio-Rad) and expression levels of genes were normalized to a reference gene β-actin. The primer pair for CXCR5 is forward, 5′-ACTCCTTACCACAGTGCACCTT-3′; and reverse, 5′-GGAAACGGGAGGTGAACCA-3′. Primers for BCL6 are forward, 5′-CACACCCGTCCATCATTGAA-3′; and reverse, 5′-TGTCCTCACGGTGCCTTTTT-3′. Primers for IL-17A are forward, 5′-GGCCCTCAGACTACCTCAAC-3′; and reverse, 5′-AGCTTCCCAGATCACAGAGG-3′. Primers for β-actin are forward, 5′-GACGGCCAGGTCATCACTATTG-3′; and reverse, 5′-AGGAAGGCTGGAAAAGAGCC-3′. Naïve control CD4^+^ cells were obtained from unimmunized wild-type splenocytes.

### Histology

Spinal cords and brains were fixed in 4% paraformaldehyde in PBS, paraffin embedded, cut, and stained with H&E and Luxol Fast Blue, according to standard protocols. For fluorescent microscopy, mice were perfused with PBS followed by 4% paraformaldehyde. Spinal cords were fixed in 4% paraformaldehyde for 4 h then placed in 20% sucrose for 48 h. Samples were embedded in OCT Compound (Sakura Finetek) and cryosectioned (7 µm) on the coronal plane. Slides were blocked with 5% normal donkey serum for 1 h and stained overnight with anti-B220 (1:400) (BioLegend, clone RA3-6B2) and anti-CD3 (1:250) (Abcam, ab5690). Slides were stained with secondary antibodies AlexaFluor 488 donkey anti-rat (Life Technologies) and AlexaFluor 546 donkey anti-rabbit (Invitrogen) for 1 h and counterstained with DAPI (Life Technologies, ProLong Diamond Antifade Mountant with DAPI). Images were collected using a Ziess LSM-710 Confocal.

### MOG Recall Assay

At the peak of TH17-EAE, spleens were collected and mechanically disrupted to generate a single-cell suspension. The cells were cultured at 2.5 × 10^6^ cells/ml for 72 h and stimulated with 0 or 10 µg/ml MOG_35–55_ in complete RPMI media. On Day 3, supernatants were collected, and IL-17 was measured by ELISA (eBiosciences).

### Flow Cytometry

The following surface antibodies were used for flow cytometry;αCD4-PECy7 or ef450 (GK1.5), αPD1-ef450 (J43), αGL7-eF450 (GL-7),αIgM-eF450 (II/41), αB220-PE (RA3-6B2), and Strepta-vidin-PE or PECy7 were purchased from eBiosciences. αPD1-APC (EH12.2H7), αIgD-PE (11-26c.2a), αCD19-PerCPCy5.5 or FITC (6D5), αCD19-PerCPCy5.5 or FITC (6D5), αB220-AF488 (RA3-6B2), αCD45.1-BV711 (A20), αCD45.2-BV605 (104), and αICOS-AF488 (C398.4A) were purchased from BioLegend. αCXCR5-Biotin was purchased from BD Biosciences.

To detect intracellular cytokines, cells were stimulated with PMA/ionomycin along with GolgiStop for 3 h at 37°C in RPMI. They were then permeabilized with Cytofix/Cytoperm (BD Biosciences) and stained with αIL-17A-FITC (TC11-18H10.1), or IL-21R/Fc chimera (R&D Systems) followed by PE-conjugated affinity-purified F(ab′)2 fragment of goat anti-human Fcγ Ab (Jackson ImmunoResearch Laboratories).

All flow cytometry data were acquired on a BD LSRII or BD FACSAriaIIIu and analyzed with FlowJo software.

### Statistics

Data are presented as means ± SEM and statistical significance was determined using a two-tailed Mann–Whitney test, Student’s *t*-test, or Kruskal–Wallis test when more than two groups were analyzed. Correlation was assessed by linear regression with calculated *R*^2^ and significance. For all data sets, differences were considered statistically significant for *p* < 0.05. All statistical analyses were made using Prism 7 (GraphPad).

## Results

### B Cells Accumulate into the CNS of TH17-EAE Mice and Contribute to Disease Severity

**Figure 1:**
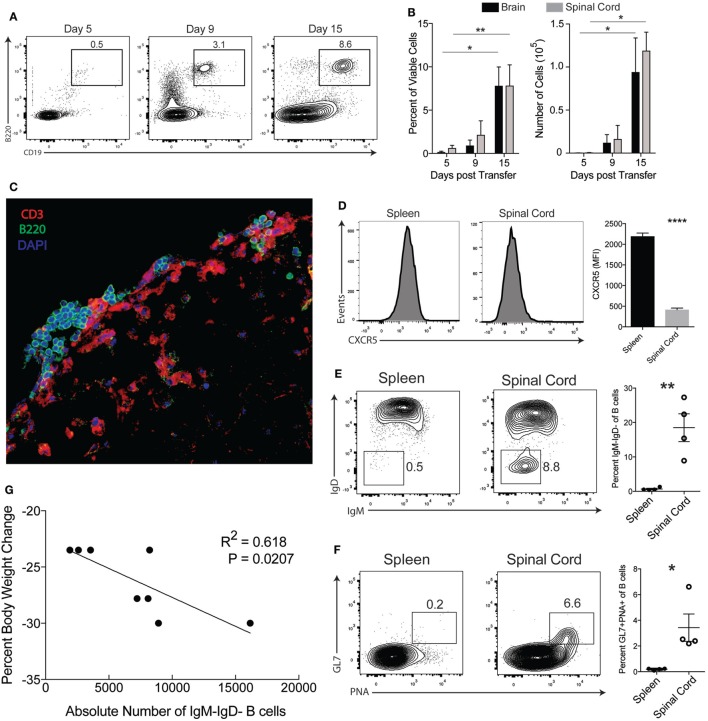
B cell responses occur in the central nervous system of TH17-EAE. **(A)** Representative flow cytometry plots of the percentage of viable B220^+^CD19^+^ B cells in the spinal cords of TH17-experimental autoimmune encephalomyelitis (EAE) at 5, 9, and 15 days post-transfer of TH17 cells. Gated on total viable cells. **(B)** Percentage of total viable cells and absolute number of B cells infiltrating the spinal cord and brain of TH17-EAE. Statistical significance from Day 5 to Day 15 was determined using Kruskal–Wallis test (**p < 0.01, *p < 0.05). Data are from one experiment representative of two experiments (*N* = 3 mice per group/experiment). **(C)** Representative image of a spinal cord section from TH17-EAE stained with anti-CD3 Ab (red), anti-B220 Ab (green), and DAPI (blue). **(D)** Expression levels of CXCR5 (measured by mean fluorescent intensity) on viable B220^+^CD19^+^ B cells in spleen and spinal cord tissue was measured by flow cytometry. Tissues were collected at the peak of disease. Statistical significance was determined using Student’s *t*-test (****p < 0.0001) and *N* = 5. **(E)** Representative flow cytometry plots of CD19^+^IgM^−^IgD^−^ class-switched B cells in the spleen and spinal cord of mouse with TH17-EAE. Gated on viable B220^+^CD19^+^ B cell population. Percentage of class-switched B cells of the total CD19^+^ B cell population in the spleens and spinal cords of mice with TH17-EAE. Statistical significance from was determined using Student’s *t*-test (**p < 0.01) and *N* = 4. **(F)** Representative flow cytometry plots of CD19^+^GL7^+^PNA^+^ germinal center B cells in the spleen and spinal cord of mouse with TH17-EAE. Gated on viable CD19^+^ B cell population in the spleens and spinal cords of mice with TH17-EAE. Statistical significance was determined using Student’s *t*-test (**p < 0.01) and *N* = 4. **(G)** Correlation between CD19^+^IgM^−^IgD^−^ class-switched B cells and decrease in body weight at the peak of disease. Statistically significant correlations were determined using linear regression and *N* = 8.

Recent studies have linked the TH17 pathway with the formation of GCs in spleens and ectopic B cell follicles at sites of inflammation (25, 26). Specifically in EAE, it was reported that TH17-EAE mice develop ectopic follicles and GC-like structures in their CNS tissues (4). For our study, we first sought to verify B cell infiltration is a feature of our TH17-EAE model; a model where we expand myelin-specific TH17 cells from MOG_35–55_-immunized mice with IL-23 and anti-IFNγ, and transfer these donor cells into healthy recipient mice (23). We assessed CNS tissue for B cells at 5, 9, and 15 days post-transfer, which correspond to before disease onset, early disease, and peak disease, respectively. We found an increasing number of B cells infiltrating the spinal cords and brains as EAE disease progressed with the highest numbers of B cells found at the peak of disease (Day 15 post-transfer of TH17 cells) (Figures 1A,B). We performed immunohistochemistry on frozen spinal cord sections and observed that B cells cluster in the meningeal areas of the spinal cord in close proximity to CD3^+^ T cells (Figure 1C). We next characterized the phenotype of the B cells in the CNS of TH17-EAE mice at the peak of disease. CXCR5 expression was significantly decreased on B cells in the CNS tissue compared to B cells in the spleen (Figure 1D). In addition, we observed that a greater percentage of the total B cell population (CD19^+^B220^+^) in the spinal cord was class-switched (IgM^−^IgD^−^) and had a GC phenotype (GL7^+^PNA^+^) compared to the B cell population in the spleens (Figures 1E,F). Strikingly, we found at the peak of disease that the number of class-switched B cells correlated with increased weight-loss in mice, which is indicative of greater disease severity (Figure 1G). These data confirm the previous reports showing that B cell responses occur in the CNS during TH17-EAE and we now show that they are positively correlated with increased disease severity.

**Figure 2:**
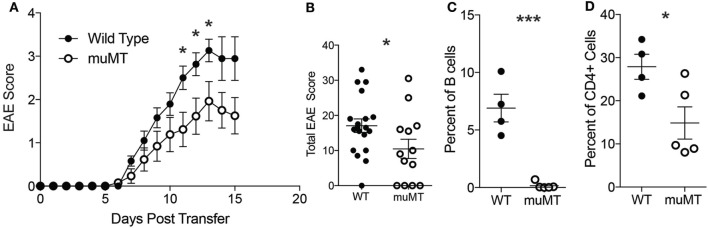
B cell deficient mice have reduced TH17-experimental autoimmune encephalomyelitis (EAE) disease severity. **(A)** Daily disease scores of TH17-EAE were compared in C57BL/6 mice and μMT. *N* = 20–21 mice per group compiled from three independent experiments. Statistical significance at each day was determined using a Mann–Whitney test (*p < 0.05). **(B)** The cumulative disease scores from (days 0 to 15) were compared in the C57BL/6 and μMT mice. Statistical significance was determined using a Mann–Whitney test (*p < 0.05). At the peak of disease, percent of viable cells which were **(C)** CD19^+^B220^+^ B cells and **(D)** CD4^+^ T cells in C57BL/6 and μMT mice spinal cords were assessed by flow cytometry. *N* = 4–5 mice per group were analyzed. Statistical significance from was determined using Student’s *t*-test (*p < 0.05, ***p < 0.0001).

Because we observed B cells infiltrating into the CNS in our TH17-EAE model, we next examined whether B cells contribute to disease progression in our disease model. To answer this question, we compared the development of TH17-EAE in wild-type mice (WT) with its development in B cell-deficient mice (μMT). We found that both WT and μMT mice developed disease, however, the μMT mice had significantly lower scores compared to WT mice (Figures 2A,B). As expected, μMT mice were almost completely devoid of spinal cord-infiltrating B cells (Figure 2C), but we also found that the μMT mice had fewer CD4^+^ T cells infiltrating the CNS compared to WT mice (Figure 2D). Taken together, these data demonstrate that B cells are promoting TH17-EAE disease severity and suggest that there is cooperation between B cells and T cells driving this pathology.

### TFH Cells Infiltrate the CNS of TH17-EAE Mice

**Figure 3:**
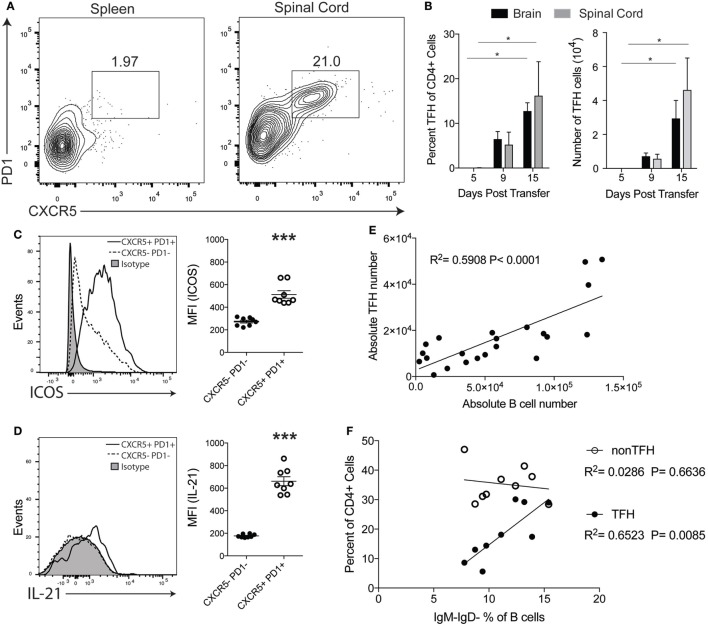
CXCR5^+^ TFH cells are present in the central nervous system (CNS) of TH17-experimental autoimmune encephalomyelitis (EAE) mice. **(A)** Representative flow cytometry plots of CXCR5^+^PD1^+^CD4^+^ T cells in the spleens and spinal cords of mice with TH17-EAE mice at Day 15 post-transfer. Gated on the total viable CD4^+^ cell population. **(B)** Percentage of CD4^+^ cells and absolute number of CXCR5^+^PD1^+^CD4^+^ T cells infiltrating the spinal cord and brain of TH17-EAE. Statistical significance from Day 5 to Day 15 was determined using Kruskal–Wallis test (*p < 0.05). Data are from one experiment representative of two experiments (*N* = 3 mice per group/experiment). **(C)** Expression levels of ICOS (measured by mean fluorescent intensity) in CXCR5^+^PD1^+^CD4^+^ T cells and CXCR5^−^PD1^−^CD4^+^ T cells was determined by flow cytometry. Statistical significance was determined using Student’s *t*-test (***p < 0.0001) and *N* = 8. **(D)** Expression levels of intracellular IL-21 (measured by mean fluorescent intensity) in CXCR5^+^PD1^+^CD4^+^ T cells and CXCR5^−^PD1^−^CD4^+^ T cells was determined by flow cytometry. Statistical significance was determined using Student’s *t*-test (***p < 0.0001) and *N* = 8. **(E)** Correlation between TFH and B cell numbers was assessed at the peak of disease. Statistically significant correlations were determined using linear regression. Data are compiled from two experiments (*N* = 10 mice). **(F)** Correlation between the percent of CD19^+^B220^+^IgM^−^IgD^−^ class-switched B cells (of total CD19^+^B220^+^ population) and the percent of CXCR5^+^PD1^+^CD4^+^ T cells and CXCR5^−^PD1^−^CD4^+^ T cells (of total CD4^+^ population) in the CNS tissues was assessed at the peak of disease. Statistically significant correlations were determined using linear regression. Data are compiled from two experiments (*N* = 10 mice).

TFH cells are primarily found in follicles of the spleens and lymph nodes during an immune response and facilitate B cell activity in the GC (20). Because we found that T cells are in close proximity to B cells in the CNS of TH17-EAE (Figure 1C), we speculated that these T cells are functioning as TFH cells and are providing help to B cells. Therefore, we assessed the frequency and numbers of CNS-infiltrating T helper cells that co-express the TFH markers CXCR5 and PD1 in mice with TH17-EAE. At the peak of disease, we found that TFH cells comprise 16.2 ± 7.6% of the total CD4^+^ T cell population within the spinal cords of mice with TH17-EAE (Figures 3A,B). In contrast, we found very few TFH cells in the spleens of these TH17-EAE mice (Figure 3A). We next examined the kinetics of TFH cell infiltration into the CNS tissue. Similar to what we observed with B cells (Figure 1B), we found that the brain and spinal cord tissue had increases in the TFH cell population as disease developed in these mice (Figure 3B). We also characterized other molecules on the infiltrating T helper cells and found that the CXCR5^+^PD1^+^CD4^+^ T cells express higher levels of ICOS and the cytokine IL-21, molecules associated with TFH function, compared to CXCR5^−^PD1^−^CD4^+^ T cells (Figures 3C,D).

We next assessed whether CXCR5^+^PD1^+^ TFH cells were correlated with B cell infiltration and activity in the inflamed CNS of mice with TH17-EAE. At the peak of disease, we found that the numbers of B cells within the CNS are positively correlated with the numbers of TFH cells (Figure 3E). Furthermore, we found there is a significant positive correlation between the percent of CXCR5^+^PD1^+^ TFH cells (in the CD4^+^ population) with class-switched B cells (in the total B cell population) in the CNS tissue (Figure 3F). Interestingly, this correlation with class-switched B cells was not observed with other CD4^+^ T cell subsets (CXCR5^−^PD1^−^CD4^+^) which indicates the correlation between TFH cells and class-switched B cells is not due to a general increase of infiltrating CD4^+^ T cells in the CNS. Instead, these correlative data suggest that there is a functional interaction between B cells and TFH cells specifically in the CNS that may promote disease progression in this model of EAE.

### TFH Cells Constitute a Second Wave of CNS-Infiltrating T-Cells

**Figure 4:**
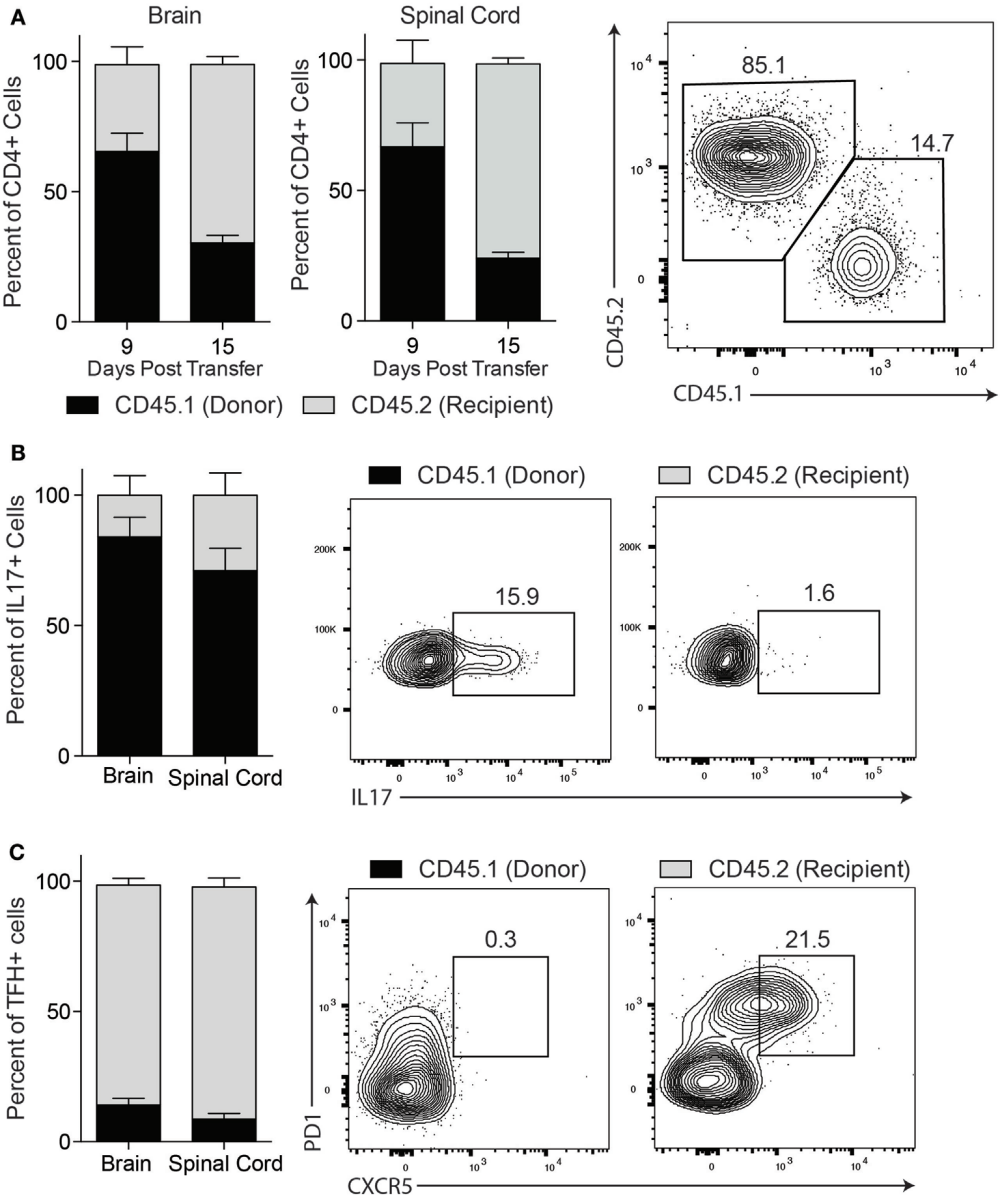
TFH cells constitute a second wave of central nervous system-infiltrating T cells and are predominantly recipient derived. TH17-experimental autoimmune encephalomyelitis was induced in wild type CD45.2^+^ mice with TH17 cells derived from CD45.1^+^ donor mice. **(A)** At day 9 and day 15 post transfer of TH17 cells, spinal cord infiltrating cells were isolated and assessed for the percentage of CD4^+^ gated cells that were derived from donor (CD45.1) or recipient (CD45.2) mice. In the spinal cord at day 15 post transfer, the percentage of **(B)** IL-17^+^CD4^+^ T cells and **(C)** CXCR5^+^PD1^+^CD4^+^ TFH cells that were donor-derived (CD45.1^+^ gated) or recipient-derived (CD45.2^+^ gated) was assessed. Data are from one experiment representative of two experiments (*N* = 3 mice per group/experiment).

To better understand the development of these infiltrating TFH cells in the CNS tissue, we wanted to first determine their origin. We generated myelin-specific TH17 cells from congenic CD45.1^+^ mice and transferred these cells into CD45.2^+^ recipient mice. We found that early in disease development (Day 9) the majority of CD4^+^ T cells in the CNS tissue are donor-derived CD45.1^+^, but by the peak of disease (Day 15) we found the recipient-derived CD45.2^+^ cells comprise the majority of the CD4^+^ T cells in the CNS (Figure 4A). This shift indicates the transferred cell population traffics into the CNS first and is followed by a second wave of immune cells derived from the recipient mouse. We next determined the phenotype of the donor and recipient cells infiltrating the CNS. At Day 15, we observed that IL-17-producing cells in the CNS were almost entirely donor-derived (Figure 4B). Conversely, we found that the majority of the TFH cells in the CNS were derived from the recipient animals (Figure 4C). These data demonstrate that TFH cells are not derived from the donor TH17 cell population, but constitute a second wave of infiltrating cells that are derived from the recipient mouse.

### TFH Cells Are Not Effective Inducers of EAE

**Figure 5:**
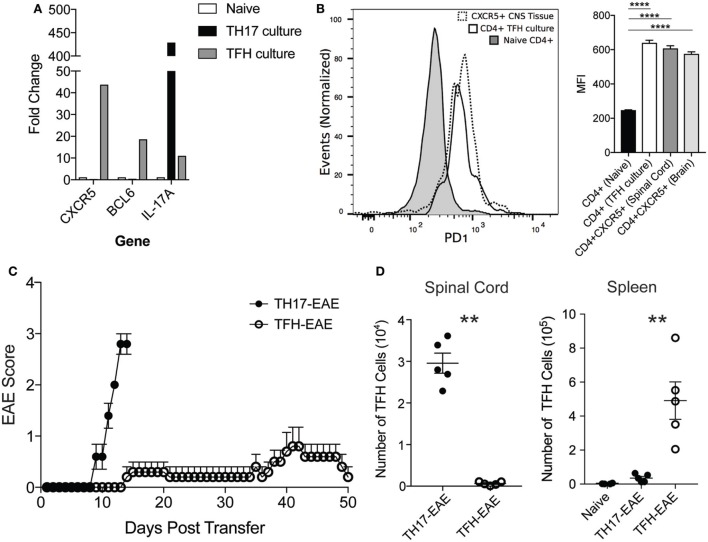
Transfer of myelin-specific TFH cells does not induce robust experimental autoimmune encephalomyelitis (EAE). Spleen cells from donor mice were cultured for 3 days in the presence of MOG_35–55_ under TH17 or TFH-polarizing culture conditions. **(A)** After culturing CD4^+^ T cells were isolated by MACS beads and assessed for mRNA expression of CXCR5, BCL6, and IL-17 by quantitative RT-PCR. **(B)** Expression levels of PD1 (measured by mean fluorescent intensity) in CXCR5^+^CD4^+^ T cells from the spinal cord, CD4^+^ T cells from TFH culture, and naïve CD4^+^ T cells was determined by flow cytometry. Statistical significance was determined using one-way ANOVA (****p < 0.0001) and *N* = 3 samples per group. **(C)** The cultured TH17 and TFH cells were then transferred into healthy C57BL/6 mice and EAE disease was monitored daily up to 50 days (Note: TH17-EAE mice were sacrificed at Day 13 due to animal welfare concerns). **(D)** Numbers of TFH cells in the spinal cords and spleens from mice with TFH-EAE and TH17-EAE were assessed by flow cytometry. TFH numbers in the spleens of naïve mice were also measured. Statistical significance was determined using Student’s *t*-test (**p < 0.01) and *N* = 5 mice per group.

Our data demonstrate that TFH cells comprise a distinct subset of immune cells in the CNS of mice with TH17-EAE, which are correlated with increased B cell infiltration and disease activity. We next questioned whether TFH cells themselves are capable of migrating into the CNS and inducing EAE without the transferred TH17 cell activity. We differentiated myelin-specific TFH cells (IL-6, IL-21, anti-IFNγ, anti-IL-4, anti-TGFβ) (24) and myelin-specific TH17 cells (IL-23 and anti-IFNγ) (23) and compared the ability of these cells to infiltrate into the CNS and induce EAE in healthy recipient mice. After *in vitro* culturing, we verified that the TFH-polarizing and TH17-polarizing conditions yielded the expected T cell phenotypes. We found that the TFH cells expressed high levels of BCL6 and CXCR5 compared to naïve T helper cells and TH17 cells. Conversely, the TH17 cells expressed high IL-17A compared naïve T helper cells and TFH cells (Figure 5A). Additionally, we measured PD1 expression on the cultured cells and found *in vitro*-derived TFH cells had PD1 levels comparable to *in vivo*-derived TFH cells in the CNS. This was significantly elevated compared to naïve CD4^+^ T cells (Figure 5B). We then transferred the TFH or TH17 cells into healthy recipient mice. In contrast to the myelin-specific TH17 cells that induced severe EAE by Day 13, we found that the myelin-specific TFH cells did not induce severe disease even by 50 days post-transfer of cells (Figure 5C). In addition, we found that TFH cells did not traffic to the CNS of mice receiving TFH cells; however, we did find substantial numbers of TFH cells homing to the spleen of these mice (Figure 5D). These data demonstrate that TFH cell alone do not initiate EAE disease but require TH17 cells to gain entry to the CNS to and promote EAE disease severity.

### CXCL13 Antibody Treatment Inhibits EAE

**Figure 6:**
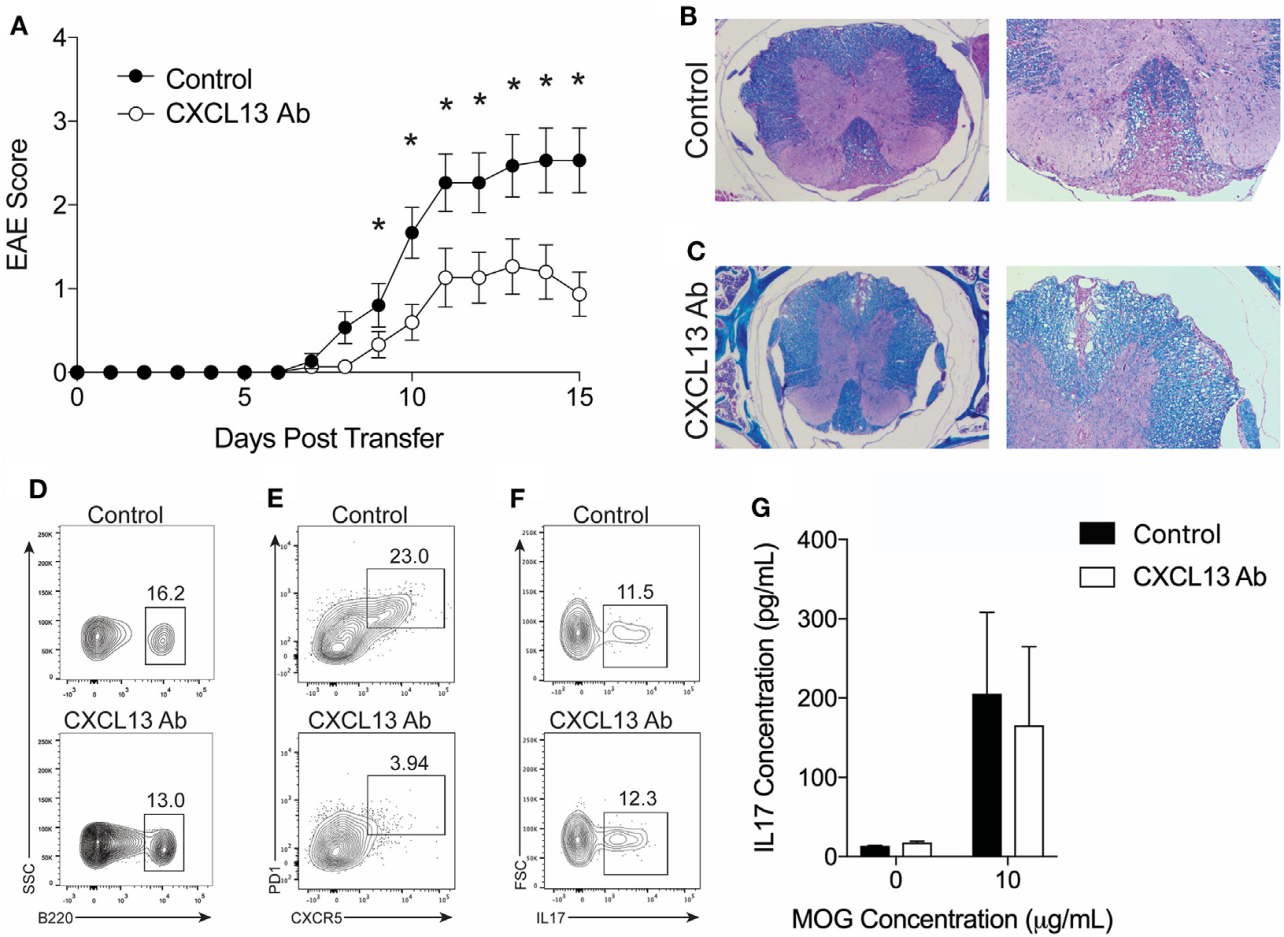
CXCL13 antibody treatment protects against TH17-experimental autoimmune encephalomyelitis (EAE) in wild-type mice. TH17-EAE was induced in C57BL/6 mice and treated with either anti-CXCL13 or an isotype control twice a week beginning at Day 0 post-transfer of TH17 cells. **(A)** Disease score was monitored. Data were compiled from three experiments and statistical significance at each day was determined using a Mann–Whitney test (*p < 0.05, *N* = 15 total mice per treatment group). Histological analysis of representative spinal cord sections stained with H&E and Luxol Fast Blue in mice treated with **(B)** isotype control or **(C)** anti-CXCL13. Representative FACS plots of the percentage of spinal cord-infiltrating **(D)** B220^+^ B-cells (in the viable cell gate), **(E)** CXCR5^+^PD1^+^ TFH cells (in the CD4^+^ cell gate), and **(F)** IL-17^+^ cells (in the CD4^+^CD44^+^ cells gate) in mice treated with anti-CXCL13 or isotype control. **(G)** MOG-specific IL-17 responses in the spleen were assessed through ELISA (*N* = 3 mice per group).

The data described above led us to hypothesize that TFH cells require a TH17 response to gain entry in the CNS, and, once in the CNS, the TFH cells facilitate the inflammatory response and contribute to disease severity. To address this hypothesis, we assessed the clinical effects of blocking TFH cell trafficking in TH17-EAE. CXCL13 is a chemokine which binds to CXCR5 and acts on both TFH cells and B cells to form GCs in the spleen during an immune response (27). Recent studies have shown that CXCL13 contributes to diseases driven by TH17 activity (28, 29). We treated TH17-EAE induced in C57BL/6 mice with anti-CXCL13 or an isotype control twice a week for the duration of the experiment. We observed that the mice treated with anti-CXCL13 had significantly attenuated disease compared to isotype control-treated mice (Figure 6A). Histology was performed on spinal cords to confirm the EAE scores was measured (Figures 6B,C). Mice treated with an isotype control had large inflammatory demyelinated lesions in the spinal cords, whereas the anti-CXCL13 treated mice had smaller inflammatory lesions with less demyelination.

We next assessed whether the anti-CXCL13 treatment altered the composition of T cell and B cells infiltrating the CNS. We found that the percentage of B cells, out of the total cell population infiltrating the spinal cord, was similar in the anti-CXCL13 treated mice (9.34 ± 1.55%) compared to isotype control-treated mice (8.95 ± 2.65%) (Figure 6D). We found decreased percentages of CNS-infiltrating TFH cells within the CD4^+^ cell population in the anti-CXCL13-treated mice (5.54 ± 3.7%) compared to control mice (15.5 ± 2.6%), with *p*-values nearing statistical significance (*p* = 0.0732) (Figure 6E). In addition, we found that the TH17 cells were not affected by anti-CXCL13 treatment. Percentages of IL-17^+^ cells of the infiltrating CD44^+^CD4^+^ T cell were similar in the anti-CXCL13 treated mice (9.1 ± 4.6%) and control mice (8.6 ± 1.9%)(Figure 6F). We also found no change in the MOG-specific IL-17 response in the spleen (Figure 6G).

**Figure 7:**
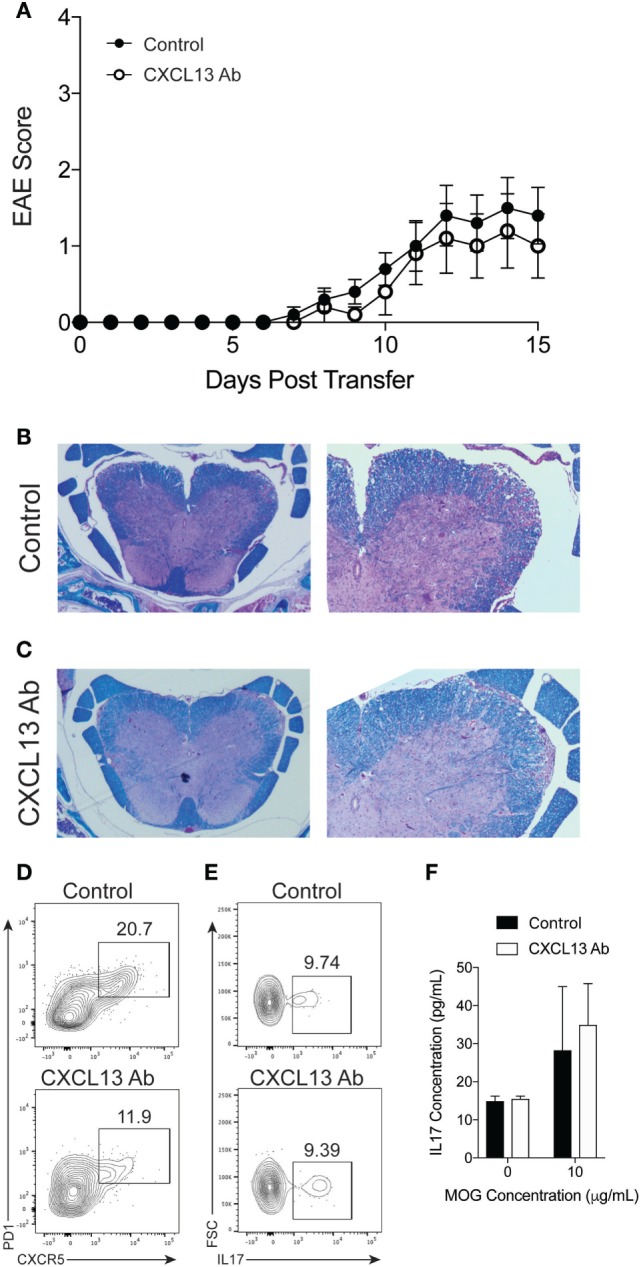
CXCL13 antibody treatment is not effective protection against TH17-experimental autoimmune encephalomyelitis (EAE) in B cell-deficient mice. TH17-EAE was induced in μMT mice and treated with either anti-CXCL13 or an isotype control twice a week beginning at Day 0 post-transfer of TH17 cells. **(A)** Disease score was monitored. Data were compiled from three experiments and statistical significance at each day was determined using a Mann–Whitney test (*N* = 10 total mice per treatment group). Histological analysis of representative spinal cord sections stained with H&E and Luxol Fast Blue in mice treated with **(B)** control antibody or **(C)** anti-CXCL13. Representative FACS plots of the percentage of spinal cord-infiltrating **(D)** CXCR5^+^PD1^+^ TFH cell (in the CD4^+^ cell gate), and **(E)** IL-17^+^ cells (in the CD4^+^CD44^+^ cell gate) of mice treated with anti-CXCL13 or isotype control. **(F)** MOG-specific IL-17 responses in the spleen were assessed through ELISA (*N* = 3 mice per group).

Finally, we determined whether B cells are required for the efficacy of anti-CXCL13 treatment. We induced TH17-EAE in μMT mice and compared the effect of anti-CXCL13 treatment on the severity of EAE. As we observed in untreated mice (Figure 2), isotype control-treated μMT mice had less severe clinical scores compared to isotype control-treated WT mice (mean maximum scores for μMT = 1.6 ± 0.40 and WT = 2.6 ± 0.37; *p* = 0.047). This was further confirmed by histology (Figures 6B and 7B). In contrast to WT mice, however, we observed that anti-CXCL13 treatment had no effect on paralysis, infiltration, or demyelination of μMT mice (Figures 7A–C). Similar to wild-type mice, we observed a trend toward a decreased TFH population in anti-CXCL13-treated mice compared to isotype control-treated mice (Figure 7D). We also found that anti-CXCL13 treatment had no effects on the TH17 population infiltrating the CNS nor in the MOG-specific IL-17 response in the spleen (Figures 7E,F).

The precise mechanism behind the efficacy of the anti-CXCL13 treatment still needs to be fully assessed. As we found that B cells have reduced CXCR5 expression in the inflamed CNS (Figure 1D) and that anti-CXCL13 treatment reduced CNS-infiltrating TFH cells but not B cells (Figures 6D,E), we speculate that blocking CXCL13 directly disrupts a critical function of TFH cells in promoting an inflammatory B cell response within the CNS of mice with TH17-EAE.

## Discussion

Previous studies have demonstrated a fundamental link between TH17 cells and B cell responses in autoimmune disease (30). It has been shown that a deficiency in IL-17 results in reduced GC formation, lowered antibody secretion, and ineffective B cell chemotaxis in the spleens of autoimmune mice (29). In EAE, the transfer of myelin-specific TH17 cells in mice induces high numbers of CNS-infiltrating B cells and ectopic follicles in the CNS tissue (4). Prior to our current study, it was unclear how TH17 cells drive disease or B cell responses in the CNS of these mice. Our current study suggests that TFH cells cooperate with TH17 cells to induce inflammatory B cell responses in the CNS and increase disease severity. Our data demonstrate that the CXCR5^+^ TFH cells in abundance within the CNS of TH17-EAE are highly correlated with B cell activity and severity of disease. Furthermore, we found that inhibition of TFH cells in the CNS with an anti-CXCL13 antibody treatment effectively reduces the severity of TH17-mediated EAE.

One question we addressed was: from what lineage do the TFH cells originate? A recent report revealed that TH17 cells differentiate into TFH cells in the Peyer’s patches and provide help for a B cell response in the gut (31). Interestingly, they showed that IL-23 was indispensable for the maintenance of the TH17 phenotype in the gut and that IL-23 blocked their differentiation into TFH cells. The results in our EAE model reinforce this relationship. When we transferred IL-23-stimulated TH17 cells to induce TH17-EAE, we found that these donor cells remain IL-17-producing cells in the CNS and do not take on a TFH phenotype. Instead, we found that the CNS-infiltrating TFH cells are derived from the recipient mice and require an inflammatory TH17 response to traffic into the CNS. It remains unclear what factors are driving the differentiation of the TFH cells *in vivo* and we have ongoing efforts to answer this question. However, as we only observe TFH cells within the CNS and not in the blood, spleen, or lymph nodes, we speculate that the environment of the inflamed CNS tissue harbors the factors that drive TFH differentiation.

Our study reinforces the hypothesis that B cell responses play an important role in driving severe TH17-driven autoimmunity and we now show that blocking a B cell response in the TH17-EAE model is beneficial in reducing disease. Yet, studies using EAE induced with active immunization with rodent MOG_35–55_ have shown that B cells possess both pro- and anti- inflammatory effects on disease (10, 32, 33). The seminal report, by Matsushita et al. (33), demonstrated that administration of CD20 antibody treatment before the induction of EAE exacerbated disease, whereas treatment of mice with established paralysis reduced disease. Subsequent studies have identified that IL-10 and IL-35 production are key mechanisms for the regulatory function of B cells and that IL-6 production and antigen presentation are key mechanisms for the inflammatory effects of B-cells in EAE (10, 34–36). Other studies have shown regulatory effects of B cells in EAE models induce by the transfer of myelin-specific TH1-cells (37). As CNS-infiltration of B cells is a feature of TH17-EAE and not TH1-EAE (4), B cells are likely to have apposing effects on disease activity in these two models. Considering our current data in the context of these previous reports, we speculate that the adoptive transfer TH17-EAE model bypasses the initial priming of myelin reactive T cells in the secondary lymphoid tissues. This avoids the regulatory effects B cells have on the early events of EAE induction seen by Matsushita et al. (33) and reveals the inflammatory effects that CNS-infiltrating B cells have on TH17-induced disease.

Recent reports have shown that ectopic follicles in the CNS and TFH cells in the blood are more prominent in secondary progressive MS patients compared to the relapsing-remitting version of this disease (21). Furthermore, CXCR5^+^ TFH cell numbers in blood are correlated with increased disability (21). These reports are highly suggestive that TFH cells play a key role in MS, especially during the progressive-debilitating phase of this disease. Our study in mice provides an important first step in understanding the relationship between TH17 cells, TFH cells, and B cells during neuro-inflammatory diseases like MS. These data provide strong evidence that blocking TFH function may be an effective strategy for treating MS, including patients with progressive versions of disease.

## Ethics Statement

All procedures and methods with animal experimentation were approved by the OMRF IACUC committee.

## Author Contributions

JQ, GK, AA, and RK performed experiments and analyzed results; RA and JQ designed the research and wrote the manuscript.

## Conflict of Interest Statement

The authors declare that the research was conducted in the absence of any commercial or financial relationships that could be construed as a potential conflict of interest.
